# Exercising in Polluted Areas: Study Suggests Benefits Outweigh the Health Risks of NO_2_ Exposure

**DOI:** 10.1289/ehp.123-A158

**Published:** 2015-06-01

**Authors:** Nancy Averett

**Affiliations:** Nancy Averett writes about science and the environment from Cincinnati, OH. Her work has been published in *Pacific Standard*, *Audubon*, *Discover*, *E/The Environmental Magazine*, and a variety of other publications.

Researchers have documented adverse effects of exposure to air pollution while exercising, such as reduced lung function among runners after they ran near busy highways^1^ and among cyclists after they rode along heavy traffic routes during rush hour.^2^ In this issue of *EHP*, however, investigators report that over the long-term, exposure to air pollution while exercising did not seem to reduce the beneficial health effects of physical activity on mortality risk.^3^

The authors used data from the Danish Diet, Cancer, and Health study, a prospective investigation of the relationships between diet, lifestyle, and cancer. From 1993 to 1997 the study recruited 57,053 men and women aged 50–65 years living in Aarhus and Copenhagen, the two most polluted cities in Denmark. This analysis included data for 52,061 individuals. At recruitment, participants reported time spent gardening, cycling, walking, and playing sports in the preceding year. The authors identified cancer, cardiovascular, respiratory, and diabetes mortality via a nationwide death registry.^3^

**Figure:**
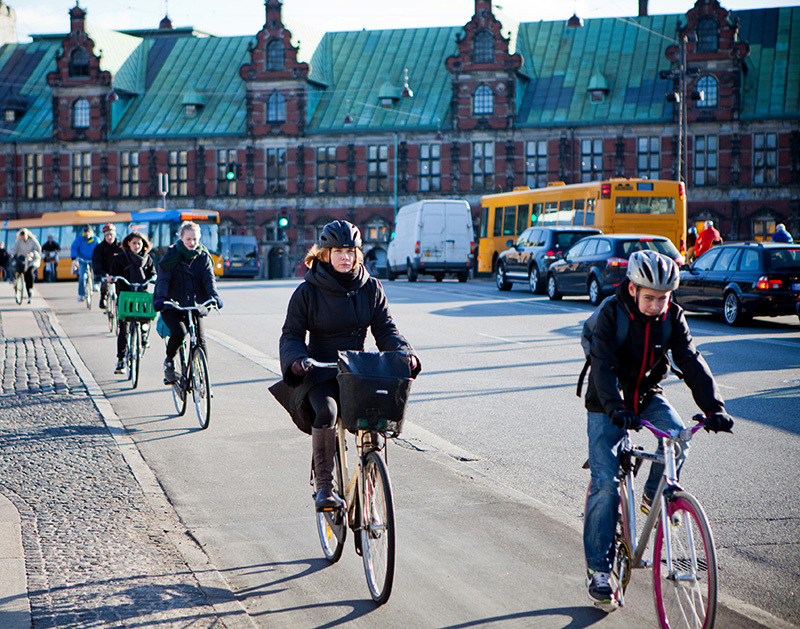
Some studies have documented short-term impacts of exercising in polluted areas, such as reduced lung function in cyclists. Over the long term, however, the beneficial effects of exercise appear to win out. © Ulrik Jantzen/Bloomberg via Getty Images

The investigators estimated air pollution exposures for each participant’s home address using mean annual concentrations from 1971 through followup, which ended 31 December 2009. They defined high exposure as the upper 25th percentile of modeled nitrogen dioxide (NO_2_) concentrations, which were calculated using the Danish AirGIS dispersion modeling system. AirGIS uses data such as traffic counts, meteorology, wind circulation, street configuration, and building position and height.^4^ The authors adjusted their model to account for a variety of lifestyle factors, such as physical activity at work, smoking status, and marital status.^3^

The authors observed a statistically significant inverse association between all the exercise categories, except for walking, and overall mortality, which did not appear to be modified by estimated exposure to NO_2_.^3^ “Our hypothesis was based on data from the short-term studies showing small damages to the lungs and heart,” says lead author Zorana Jovanovic Andersen, an associate professor at the Centre for Epidemiology and Screening at the University of Copenhagen. “We thought [such damage] would probably accumulate over time and reduce some of the benefits of exercise. But we didn’t find this.”

Instead, Andersen says, exercise seemed to offer a protective effect, with even the high-exposure air pollution category associated with a reduced risk of overall mortality of up to 25%. “So we think that the damages [to heart and lungs] are probably transitional,” she says, “and maybe they are improved by exercise.”

The study results complement recent research conducted by Michael Koehle, an associate professor of kinesiology at the University of British Columbia. Koehle and his graduate students looked at how exercise intensity affected the body’s response to air pollution, expecting to find that higher-intensity exercise would be associated with stronger effects from pollution, but instead finding the opposite.^5^

“Our research is looking at acute exposures in younger people, so this paper [by Andersen et al.], which looks at long-term effects in an older population, is very complementary,” Koehle says. “Also we looked at diesel exhaust, which is high in particulate matter, whereas the Andersen paper looked at NO_2_. So in many ways their research was quite different, yet it confirms what we were finding.”

The results also are consistent with two recent experimental studies from Barcelona, specially designed to disentangle interactions between short-term effects of air pollution and physical activity on lung and heart function. Findings from those studies suggested that in healthy subjects, benefits of physical activity outweighed the risks related to air pollution exposure.^6^^,^^7^

Limitations to the Danish study include the fact that the exercise category of playing sports was not defined, so the authors did not know if the subjects were participating in outdoor or indoor activities. In addition, NO_2_ data were limited to measurements taken near subjects’ residences and thus may not pertain to exercise performed in another location.^3^

Finally, the authors note the cohort was better educated and had a higher income compared with the general Danish population, so they may have been healthier overall. Plus, while the two Danish cities do have pollution problems, they are relatively clean compared with other cities around the world.^3^ “I think Copenhagen is comparable to northern European cities and some American cities,” Andersen says, “but not cities with several magnitudes of higher air pollution, such as in India and China.”

Despite the study’s results, Andersen says urban exercisers should still seek out less-polluted streets and green spaces if they can. She adds a word of advice to urban residents who may opt out of exercising as a way to avoid air pollution: “You still get [exposed to] air pollution if you sit at home on your sofa or drive a car along the same road. You cannot avoid air pollution by avoiding exercise, but you can avoid the health benefits of exercise.”
